# The Influence of Functional Fitness and Cognitive Training of Physical Disabilities of Institutions

**DOI:** 10.1155/2015/686498

**Published:** 2015-02-09

**Authors:** I-Chen Yeh, Chia-Ming Chang, Ko-Chia Chen, Wei-Chin Hong, Yu-Hsiung Lu

**Affiliations:** ^1^Physical Education Center, National Kaohsiung Marine University, Kaohsiung 811, Taiwan; ^2^Department of Physical Education, Health & Recreation, National Chiayi University, Chiayi 621, Taiwan; ^3^Physical Education Office, National Kaohsiung University of Hospitality and Tourism, Kaohsiung 812, Taiwan; ^4^National Sports Training Center, Sports Administration Ministry of Education, Kaohsiung 813, Taiwan

## Abstract

According to an investigation done by Taiwan Ministry of the Interior in 2013, there was more than 90% of the disability care institutions mainly based on life care. Previous studies have shown that individuals can effectively improve physical and cognitive training, improved in independent living and everyday competence. The purpose of the study was to investigate influence of the intervention program applying functional fitness and cognitive training to disabled residents in the institution. The subjects were disabled persons of a care institution in southern Taiwan and were randomly divided into training and control groups, both having 17 subjects. The age of the subjects was between 56 and 98 years with a mean age of 79.08 ± 10.04 years; the subjects of training group implemented 12 weeks of training on physical and cognitive training, while the control group subjects did not have any training program. The results revealed that subjects of the training group have significantly improved their functional shoulder rotation flexibility of left and right anterior hip muscle group flexibility of right, sitting functional balance of left and right, naming, attention, delayed recall, orientation, and Montreal cognitive assessment (MOCA). The study suggested developing physical fitness programs and physical and cognitive prescriptions for the disabled people of the institutions.

## 1. Introduction

### 1.1. With the Sharp Increase in the Aging Population, Long-Term Care Becomes More Urgent

According to the statistics of the Ministry of the Interior [1], by the end of 2011, the total number of household registration was 23,160,000, with the lowest sum total of merely 1.8% in the increased population on annual records. Based on the statistics in late 2010, there were totally 1,067 long-term care facilities and care homes for the elderly (except for Veterans homes and nursing homes) in Taiwan. 56,256 people are provided, but there are just 41,929 people living in such facilities, with the increased tendency of 74.5%. Among them, 76.9% nursing institutes were the highest, followed by 70.9% in long-term care facilities and 69.2% in care homes. Most of the long-term care facilities are in New Taipei, followed by Taipei City and Kaohsiung City [2].

### 1.2. The Physical and Mental Disabilities Also Extended the Average Life Expectancy Trend

The Ministry of the Interior [2] reported that over 1,064,000 Manual of Intellectual and Mental Disorders had been distributed since the end of June, 2010. There are 272 institutions only for people with Intellectual Disabilities Caring in Taiwan. In addition to full-day services, night accommodations, day care, and part-time services, the Ministry of the Interior also provides all kinds of respite care, short-term care, and counseling services. Comparing public and private institutions, the private ones are dispersed with the highest quantity of 189; there are 67 public-private institutions in second. As to the public organizations, there are merely 16 ones. If classified according to the types of institutes, most of them can be seen to mainly provide life care services. Furthermore, on the basis of the documents from the Ministry of the Interior [2], 386,050 people with physical disabilities cover the highest rate of 35.58% among all the people with disabilities. If differentiating based on local governments, New Taipei covered 141,849 people with the highest rate of 13.07%, and Kaohsiung covered 128,891 with the second highest rate of 11.88%. Due to the influences of decline in fertility rates and of longevity extension in Taiwan, the demographic structure gets aging in Taiwan. However, with the population growth, the people with disabilities also live longer [3], which means the time of their medical care needs to be extended and will affect the caregivers and the healthcare systems [4].

### 1.3. In Institutes the Learning Needs of the Disabilities Are Neglected, Which Goes against the Utilization of Limited Medical Sources

R. A. Kane and R. L. Kane [5] indicated that the disabilities in communities and facilities expect to improve or maintain their fundamental body functions through care services so that they can enhance their self-care abilities. As far as Weissert [6] are concerned, the subjects for long-term care are supposed to accommodate the population of all ages in institutes, noninstitutes, and households in order not to improve their body functions but to take social and mental issues into considerations. Domestic researcher Wu [7] found that the more serious the body dysfunction for elders is, the higher the demands for social services will be. As to caregivers, they have to both work and take care of the elderly in the meantime. Those who perceive heavy burden or have less care support are in high demand of social services. Instituted-care is for people with physical and mental disabilities, and it offers the services of accommodations, nursing, health care, medical treatment, individual care, pick-up services, counseling, physical therapy, occupational therapy, and so on. Moreover, it can also relieve the burden of patients' family.

### 1.4. It Has Been Confirmed That Functional Fitness and Cognitive Training Can Contribute to the Health of the General Elders or the Disabled

It has been found that aging makes changes to our body, such as muscle weakness, impaired balance, coordinating and flexibility reduction, and loss of sensation. Therefore, these combined effects will increase the risk of falls. Chang et al. [8] indicated that the improvement of the sport and activity level will help to minimize the risk of falls, especially the promotion of balance and muscle strength. Donat and Özcan [9] selected 42 subjects from a 535-resident nursing home and randomly divide them into two groups: the unsupervised group and supervised group. Exercise sessions were performed three times a week for eight weeks. As a result, in addition to balance, functional mobility, and flexibility, the supervised exercise group showed significant improvements in both strength and proprioception. The unsupervised home exercise group showed significant improvement in balance, functional mobility, and flexibility. Hence, subjects of the supervised group were more effective at reducing the risk factors related to falling among older adults living in a nursing home than those of the unsupervised subjects. One year after the start of the intervention at posttreatment. Participants in the physical and cognitive training improved in independent living and everyday competence [10]. Valenzuela and Sachdev [11] indicated that an overall risk reduction of 46% for high mental activity levels compared to low activity. Interestingly, the independent effects of education, occupational complexity, and cognitive life style were similar in magnitude. de Carvalho Bastone and Filho [12] performed a study that determined the effectiveness of a 6-month program of regular exercises with a view to the improvement of functional performance of the elderly living in a nursing home. Forty subjects aged over 60 were selected to take part in this trial to either a comparative group or an exercise group. In the end, the exercise subjects showed significant improvement in lower-limb function test, 6-minute gait velocity, knee extensors strength, and the Geriatric Depression Scale (GDS); there was no significant performance improvement in Mini-Mental State Examination (MMSE). However, the nonexercise subjects in control group showed significant decrease in lower-limb function, gait velocity, GDS, and MMSE.

### 1.5. Functional Fitness Is a Key to Aging in a Healthy Way, so It Is Supposed to Become One of the Targets in the Community of Senior Adult Education and in the Long-Term Care Institutes

The Ministry of the Interior [1] pointed out that an accumulative total of each subsidy for disabilities had reached 10.56 billion dollars from January to June in 2012, which increased 5.6% if compared to the same period in 2011.

Due to the increasing population growth in Taiwan, the expenditure of national medical care and of health as well as welfare relatively rose up. Through the research background discussion above, the inadequacy of problems and importance of the study could be examined. Domestic studies [7, 13–15] seldom investigate on the practice of multiprofessional areas. Nevertheless, most of the research methods are conducted through comparisons, telephone interviews, and face-to-face interviews while selecting residents as their subjects in the facilities. Researchers anticipate that in accordance with the results, follow-up studies and practiced can be provided in order to develop adequate exercise prescriptions and cognitive performance courses for disabilities, to help them cultivate regular exercises, the habits of life-long learning, and to achieve the prospects of active aging learning [16]. With that, despite the fact that the disabilities may stay in the facilities all the time, they still have the rights to learn, enjoy somatopsychic health promotions, and reduce the continual expense of medical care and social resources. The references are mainly based on successful aging [17].

The purpose of this study was to survey the residents in facilities as its objects are in accordance with (a) the discussion of present functional fitness and cognitive function to people with physical disabilities; (b) the significant results of functional fitness and cognitive function so that recommendations can be provided to plan courses of exercise and cognitive prescriptions for people with physical disabilities.

## 2. Method

### 2.1. Research Design Assumptions

The study aims to conduct the people with disabilities as subjects and it could be used as intervention strategies for functional fitness and cognitive training to investigate the improvements that people with physical disabilities work on functional fitness and cognitive function including back-and-forth shoulder rotator flexible degree, supine passive ankle dorsiflexion, supine passive knee straight position, hip muscles test angle, grip strength, functional leg strength and posture functional balance test and to develop the flexibility, grip strength, functional leg strength, balance, visuospatial, naming, attention, language, abstraction, delayed recall, orientation, and Montreal cognitive assessment (MOCA) of people with physical disabilities. In accordance with the conclusion of relevant studies [8–10, 12], researchers assume that values will show significant improvements.

### 2.2. Subjects

This study was conducted in a nursing home in Kaohsiung, which hosts 49 people with physical disabilities and was a Grade B nursing home evaluated by Department of Health, Executive Yuan. Those who had diseases of nervous, respiratory, and musculoskeletal disorders that went against exercise intervention were expelled from the study. According to the revision of “the degree of accreditation to people with disabilities” announced by Ministry of the Interior [18], physical disabilities were defined as “any impairment which limits the physical function of limbs due to developmental retardation, pathological changes of central or peripheral nervous system, injuries, or other congenital and acquired damage of skeletal musculature system.” This research was mainly based on the people with Manual of Intellectual and Mental Disorders. Subjects with mean age of 79.09 ± 10.40 years were randomly separated into training group and control group (with 7 males and 10 females in each group). The subjects of training group performed a 12-week and moderate-intensity exercise intervention three times a week. The exercises like warm-ups, main activities, and cool-downs last 35 minutes each time and cognitive training course intervention two times a week. The cognitive training like Cards, paired, and reading last 50 minutes each time. The subjects of control group did not get involved in any activities. Before and after the 12-week physical and cognitive training intervention, all subjects completed a pretest and a posttest, respectively, which included flexibility, grip strength, functional leg strength, balance, visuospatial, naming, attention, language, abstraction, delayed recall, orientation, and MOCA and then differences were evaluated. Data were collected in the 12 weeks from July to September in 2012. All the procedures of the physical and cognitive training intervention were not invasive and were approved by owners of the facilities. Parents of the subjects have signed the consent letters.

### 2.3. Research Projects and Tools

*(A) Detection Checklist.*

1. Flexibility: includes left and right functional rotator flexible degree, left and right foot supine passive ankle dorsiflexion, left and right foot supine passive knee straight position, and left and right hip muscles test position [19].
2. Hand grip strength test standards: in accordance with the American College of Sports Medicine [20] as the standard.
3. Functional leg strength test [21].
4. Seated functional balance test [22].
5. Montreal Cognitive Assessment (MoCA) assessment by Nasreddine et al. [23] as the standard.

*(B) Test Content*. The assessment items include (1) flexibility which was measured by rotating action flexibility on left and right shoulder, left and right foot supine passive ankle dorsiflexion position, left hip muscle test angle, and the angle of right hip muscle testing. The protractor was measured by standing participants with shoulders of spin flexibility and supine attitude of passive hip, knee, and ankle position [19]; (2) hand grip strength was measured by sitting with the elbow flexed to 90 degrees, sparing no efforts to grab the hand grippers in both hands; (3) functional leg strength was measured by standing timing meter. Subjects were required to repeatedly sit down and stand up without support in the upper extremities within 30 seconds and counted the number of times they made; (4) functional sitting balance test was measured by stretching arms to 90 degrees under independent balance conditions, and the number of times were recorded; (5) MoCA was measured with a total of 30 points (cognitive assessment scoring less than 26 points on average, the phenomenon of neurocognitive dysfunction).

Subjects in both groups were required to, respectively, complete a pretest before the 12-week physical and cognitive training intervention and a posttest after the physical and cognitive training intervention. The training group was required to carry out training interventions after the pretest.

### 2.4. Assessment Procedures of the Exercise Intervention

Physical intervention includes warm-ups, main exercises, and cool-downs. After measuring resting heart rates, the subjects of training group began to warm up for ten minutes. The physical activities are mark time (wheelchair) and static stretching including shoulder, neck, chest, back, side of the body muscles, deltoids, triceps, quadriceps, hamstring, and calf stretch [24]. Main exercises included knee embracing, towel pull, hand grasp on hand rails, wrist ball, and sit-to-stand, with the moderate exercise of 5% strength [25] every four weeks [26]. The main implements of the exercise intervention are described as follows. The subjects lay in the bed holding the left and right knee to the chest; subjects used elastic band pulling up and down (wheelchair) around the shoulders; subjects hold a gravity ball standing up from a wheelchair. After main exercises, subjects had a 10-minute cool-down, which included mark time (wheelchair) and static stretching [24] including shoulder, neck, chest, back, side of the body muscles, deltoids, triceps, quadriceps, hamstring, and calf stretch. Cognitive intervention includes teachers help, digital Rus, letters Tsuburi, memory expert, little secretary, city maps, shadow of a Kind, small forensics home, five kinds of unit mix [27].

### 2.5. Data Processing

After the Completion of 12-week physical and cognitive training interventions and data collections, SPSS for Windows 19.0 was used for descriptive statistical analysis so that researchers could acquire basic understandings of the basic variables related to demographic distribution of subjects. All variables (including flexibility, grip strength, functional leg strength and balance, visuospatial, naming, attention, language, abstraction, delayed recall, orientation, and MOCA) were considered by means of comparing the results between the subjects in both groups by statistical tests such as two-way mixed design ANOVA and* t*-test. After the 12-week physical and cognitive training intervention, the differences of the subjects in both control and training group were assessed by Paired* t*-test test. The estimation of *P* < 0.0125 reached the statistical significance.

## 3. Results

As seen in Table 1, the sex ratio between the subjects of both training group and control group to demographic survey is 10 : 7. Results for sex and age of subjects in both groups did not significantly vary in the statistical tests. Before the 12-week physical and cognitive training intervention, the subjects in both groups completed a pretest including flexibility, grip strength, functional leg strength, balance, visuospatial, naming, attention, language, abstraction, delayed recall, orientation, and MOCA. In Table 2, statistical tests such as two-way mixed design ANOVA indicated that subjects in training group had significantly more positive response than control group on functional shoulder rotation of left, functional shoulder rotation of right, anterior hip of left, anterior hip of right, functional leg strength, standing functional reach of left, standing functional reach of right and attention, after the 12-week physical and cognitive training intervention (*P* < 0.05). Comparisons of each program assessment before and after the 12-week physical and cognitive training intervention in the training group appeared in Table 3. The results revealed that after the 12-week physical and cognitive training intervention, the subjects in the training group had more preferable flexibility, balance, and MOCA than those in the control group, with significant improvement (*P* < 0.0125) of left and right functional rotator flexible degree, right hip muscles flexible degree, functional left-posture and right-posture standing balance, orientation, and MOCA assessment project. Comparisons of each program assessment before and after the 12-week physical and cognitive training intervention in the control group appeared in Table 4. The statistically evaluated results of both pretest and posttest in the control group did not reach the significant differences. That is, no significant effects emerged on any of the variables before and after the 12-week physical and cognitive training intervention in control group.

## 4. Discussion

### 4.1. The Present Situations of Functional Fitness for Subjects with Physical Disabilities in the Facilities

The results indicated that the left and right functional rotator flexible degree, left feet and right feet supine passive ankle dorsiflexion, supine passive knee straight position, the left and right hip muscles flexible degree, grip strength, functional leg strength, balance, visuospatial, naming, attention, language, abstraction, delayed recall, orientation, and MOCA of the subjects are not up to standards. Therefore, subjects need to reinforce their training on the left and right shoulders rotator flexibility, the muscles around the knee, hamstring flexibility, front left and right hip muscles flexible degree, upper and lower extremity muscle strength, balance, and cognitive training. Previous study reported that behavior change coupled with persistent regular exercise training assists flexibility, muscle, enhance cardiorespiratory fitness, and cognitive function [8, 10, 12, 28, 29]. The subjects in this study were based on nursing home life care, for they can merely afford limited physical activities. In the long run, appropriate physical and cognitive prescriptions are beneficial to physical fitness and cognitive function of the subject with physical disabilities.

### 4.2. The Improvements on Functional Fitness of the People with Physical Disabilities by Physical and Cognitive Training Intervention

In this study, the people with physical disabilities were selected as subjects to explore the improvements of physical and cognitive training intervention on the flexibility, grip strength, functional leg strength, balance, and MOCA to people with physical disabilities. The results showed that the subjects of training group had more significant performance improvements on flexibility, balance, orientation, and MOCA after the 12-week training intervention than those before training intervention; that is, the 12-week training intervention are beneficial to improve body movements and physical fitness and cognitive function for people with physical disabilities. A relevant study [9] selected 42 subjects and randomly divided them into two groups: the unsupervised group and supervised group. Exercise sessions were performed three times a week for a period of eight weeks. As a result, in addition to balance, functional mobility, and flexibility, the supervised exercise group showed significant improvements in both strength and proprioception. Static and dynamic symmetrical body-weight training was also distributed among patients with 3-month hemiplegic stroke [30]. Significant improvement in sit-to-stand performance was found and the number of falls by stroke patients decreased. Above all, subjects performing 12-week medium intensity exercise intervention spent much time on exercise intervention compared to the above studies. Subjects involved in the 12-week exercise intervention also showed significant improvements in flexibility and balance. As Naylor et al. [31] found, even a regular scheduled activity program with social activities and light physical activities (e.g., going for a walk, exercises, and stretching) improved memory functions and physiological parameters of brain function (e.g., EEG). The finding that few short- or long-term effects of either psychoeducational training or physical training were detected can be explained as follows: although physical training seems to improve the metabolic activity of the brain, the cognitive potential released cannot be actively utilized without a specific cognitive training [10]. Therefore, continuous regular training helps support physical and cognitive status of the disabled and those who are unable to live on their own and to manage their physical-mental health.

## 5. Conclusions and Recommendations

Our results suggest that long-term and day-care physical disabled residents in facilities have little time to learn reading, writing, and operations due to the group life of instituted care and the majority of their severe dependence, which is much higher than the residents in nursing homes. In addition, since the physical disabled are identified from mild to severe extents, they tend to have low self-gratification and sense of accomplishment. Considering physical and cognitive training is beneficial to health, the efficacy of physical and cognitive prescriptions will be influenced based on different kinds of disabled patients.

In summary, our study suggests that institute owners can plan some muscular strength and cognition activities in terms of environments, manpower, and so forth to support its basic metabolic rate (BMR), to improve the performance, to increase energy expenditure, reduce chronic diseases, improve quality of life, provide lagging learning areas, and draft appropriate lifelong learning courses. In other words, if the leading institutes can design individualized courses by catering to the interests and physical conditions of the physical disabled so that they can mentally and spiritually create more positive developments in the long term.

This study carried out physical and cognitive training for 12 weeks. Those who had diseases of nervous, respiratory, and musculoskeletal disorders that went against physical and cognitive intervention were expelled from the study. The body movement abilities and physiological conditions were taken into considerations when subjects with physical disabilities performed exercise interventions. Therefore, the adaptations for people with deficient body movements and physical disabilities need to be further explored. Due to the factors of manpower, resources, and so on, the subjects with physical disabilities came from only one nursing home. Secondly, during the exercise intervention, subjects had difficulties controlling their emotions. Consequently, the small sample size turned out to be another limitation. Thirdly, since blood biochemical tests were not collected, researchers were limited to infer physiological changes from people with physical disabilities.

This research is merely based on the perspectives of educators, athletes, and physical therapists. In the future, the combination of lifelong educators, doctors, clinical psychologists, clinical pharmacists, nurse practitioners, social workers, physical therapists, occupational therapists, dietetics, teachers in physical education specialty, counselors, volunteers, religious workers, medical educators, and long-term personnel is recommended to advance interdisciplinary cooperation with a view to maintaining healthy lifestyle and habits for the long-term residents with physical disabilities and to bring benefits to their lifelong learning.

## Figures and Tables

**Table 1:** Basic data.

Variables	Training	Control
	(*n* = 17)	(*n* = 17)
Gender		
Male	7	7
Female	10	10
Age (yrs)		
Mean	78.71	79.47
Standard deviation	8.74	12.09
Maximum	89.00	98.00
Minimum	56.00	57.00
Education		
None	9	8
Elementary	3	3
Junior	3	1
Senior	1	4
University	1	1

**Table 2:** Two-way mixed design ANOVA.

Items	SS	df	MS	*F*
Flexibility				
Functional shoulder rotation of left				
Training	13.24	1	13.24	.04
Timing	89.47	1	89.471	11.30
Variables test	165.24	1	165.24	20.88^*^
Functional shoulder rotation of right				
Training	21.24	1	21.24	.06
Timing	23.53	1	23.53	5.06
Variables test	39.77	1	39.77	8.56^*^
Passive ankle dorsiflexion of left				
Training	208.25	1	208.25	.83
Timing	.13	1	.13	.06
Variables test	4.25	1	4.25	2.03
Passive ankle dorsiflexion of right				
Training	237.19	1	237.19	2.14
Timing	.13	1	.13	.02
Variables test	24.72	1	24.72	3.08
Passive knee extension of left				
Training	455.53	1	455.53	1.13
Timing	2.88	1	2.88	.24
Variables test	2.12	1	2.12	.17
Passive knee extension of right				
Training	833.00	1	833.00	3.06
Timing	46.12	1	46.12	2.30
Variables test	.94	1	.94	.05
Anterior hip of left				
Training	4.77	1	4.77	.05
Timing	8.47	1	8.47	2.29
Variables test	21.24	1	21.24	5.74^*^
Anterior hip of right				
Training	.94	1	.94	.01
Timing	19.06	1	19.06	16.12
Variables test	31.12	1	31.12	26.33^*^
Strength				
Functional leg strength				
Training	3.77	1	3.77	.37
Timing	.06	1	.06	.13
Variables test	2.88	1	2.88	6.13^*^
Grip of left				
Training	87.19	1	87.19	.28
Timing	.72	1	.721	.64
Variables test	1.78	1	1.78	1.58
Grip of right				
Training	4.77	1	4.77	.02
Timing	3.77	1	3.77	.50
Variables test	19.06	1	19.06	2.52
Balance				
Standing Functional Reach of left				
Training	353.31	1	353.31	3.61
Timing	22.37	1	22.37	10.12
Variables test	58.37	1	58.37	26.39^*^
Standing Functional Reach of right				
Training	233.47	1	233.47	2.17
Timing	52.94	1	52.94	24.87
Variables test	84.94	1	84.94	39.90^*^
Montreal cognitive assessment (MoCA)				
Training	.72	1	.72	.01
Timing	1.19	1	1.19	.07
Variables test	24.72	1	24.72	1.43
Visuospatial				
Training	.13	1	.13	.04
Timing	.13	1	.13	.07
Variables test	1.78	1	1.78	.88
Naming				
Training	.06	1	.06	.09
Timing	.94	1	.94	6.02
Variables test	.06	1	.06	.38
Attention				
Training	3.31	1	3.31	1.01
Timing	.02	1	.02	.01
Variables test	12.37	1	12.37	5.64^*^
Language				
Training	.72	1	.72	.50
Timing	.37	1	.37	.68
Variables test	.72	1	.72	1.32
Abstraction				
Training	.368	1	.368	.99
Timing	.13	1	.13	.81
Variables test	.13	1	.13	.81
Delayed recall				
Training	1.47	1	1.47	.89
Timing	.53	1	.53	.55
Variables test	2.88	1	2.88	3.02
Orientation				
Training	7.78	1	7.78	6.73
Timing	10.72	1	10.72	15.389
Variables test	2.49	1	2.49	3.57

^*^ *P* < 0.05, two-way mixed design ANOVA.

**Table 3:** Comparisons of each program in training group.

Variables	Before		After		*t*-value
	Mean	SD	Mean	SD	
Flexibility					
Functional shoulder rotation of left	56.12	15.72	50.71	15.00	4.33^*^
Functional shoulder rotation of right	55.06	11.51	52.35	13.21	3.19^*^
Passive ankle dorsiflexion of left	9.65	12.52	10.06	12.13	−0.68
Passive ankle dorsiflexion of right	13.24	8.41	14.35	7.36	−1.08
Passive knee extension of left	−49.35	15.44	−49.41	15.73	0.06
Passive knee extension of right	−48.71	14.47	−46.82	11.65	−1.27
Anterior hip of left	−20.94	7.55	−19.29	7.10	−2.04
Anterior hip of right	−21.76	8.92	−19.35	8.09	−5.86^*^
Strength					
Functional leg strength	1.94	2.59	2.29	2.57	−1.85
Grip of left	11.94	15.26	12.47	15.34	−1.45
Grip of right	12.41	12.70	13.00	12.84	−1.57
Balance					
Standing functional reach of left	14.65	7.40	17.65	8.67	−7.00^*^
Standing functional reach of right	15.18	7.07	19.18	8.44	−7.11^*^
Montreal cognitive assessment (MoCA)	9.82	6.21	11.29	6.87	−4.27^*^
Visuospatial	0.53	1.23	0.94	1.75	−1.60
Naming	1.82	0.73	2.12	0.70	−2.58
Attention	1.76	2.05	2.65	1.46	−2.43
Language	0.88	1.05	0.94	1.03	−0.57
Abstraction	0.35	0.49	0.53	0.62	−1.85
Delayed recall	1.71	1.40	2.29	0.77	−2.58
Orientation	2.65	1.00	3.82	1.01	−4.29^*^

^*^ *P* < 0.0125, Paired *t*-test.

**Table 4:** Comparisons of each program in control group.

Variables	Before		After		*t*-value
	Mean	SD	Mean	SD	
Flexibility					
Functional shoulder rotation of left	53.88	12.70	54.71	11.98	−1.50
Functional shoulder rotation of right	52.41	13.96	52.76	13.53	−0.58
Passive ankle dorsiflexion of left	6.65	9.76	6.06	10.30	1.66
Passive ankle dorsiflexion of right	10.71	7.34	9.41	7.69	1.43
Passive knee extension of left	−54.18	14.35	−54.94	11.72	0.55
Passive knee extension of right	−55.47	9.98	−54.06	11.83	−0.89
Anterior hip of left	−20.35	7.30	−20.76	7.32	1.51
Anterior hip of right	−20.65	3.43	−20.94	3.70	0.89
Strength					
Functional leg strength	2.82	2.30	2.35	1.73	1.73
Grip of left	10.00	9.18	9.88	8.95	0.32
Grip of right	14.00	13.99	12.47	10.61	1.19
Balance					
Standing Functional Reach of left	11.94	5.86	11.24	5.97	1.22
Standing Functional Reach of right	13.71	6.96	13.24	7.06	1.10
Montreal cognitive assessment (MoCA)	10.82	5.47	9.88	5.11	0.47
Visuospatial	0.94	1.75	0.71	1.57	0.37
Naming	1.94	0.56	2.12	0.49	−1.14
Attention	2.18	1.67	1.35	1.37	1.33
Language	1.29	0.92	0.94	0.97	1.03
Abstraction	0.29	0.47	0.29	0.47	0.00
Delayed recall	1.82	1.24	1.59	1.06	0.57
Orientation	2.35	1.00	2.76	0.83	−1.38

^*^ *P* < 0.0125, Paired *t*-test.
